# Clinical Aspects of Mental Imagery in Alzheimer’s Disease-Related Dementia: A Review of Cognitive, Motor, and Emotional Interventions

**DOI:** 10.3390/brainsci15030223

**Published:** 2025-02-21

**Authors:** Anna Christakou, Marousa Pavlou, George Stranjalis, Vasiliki Sakellari

**Affiliations:** 1 Department of Physiotherapy, School of Health Sciences, University of West Attica, 12243 Athens, Greece; vsakellari@uniwa.gr; 2 Laboratory of Biomechanics, Department of Physiotherapy, School of Health Sciences, University of Peloponnese, 23100 Sparta, Greece; 3 Centre for Human & Applied Physiological Sciences, King’s College, London WC2R 2LS, UK; marousa.pavlou@gmail.com; 4 Department of Neurosurgery, University of Athens, Evangelismos Hospital, 10676 Athens, Greece; stranjal@otenet.gr

**Keywords:** mild cognitive impairment, dementia, Alzheimer’s disease, mental imagery, cognitive ability, balance, functional status, strength, depression, anxiety, quality of life

## Abstract

The present review describes the use and effectiveness of mental imagery in Alzheimer’s disease-related dementia. Six databases were thoroughly searched from January 2010 to December 2024. Different types of studies were retrieved and reviewed for imagery of the motor, cognitive, and emotional states and quality of life of the elderly with dementia. Although the scarce results showed the positive effect of mental imagery to the every-day life of older adults with dementia, more research should be conducted with larger homogenous samples and more valid tools. Future recommendations are provided.

## 1. Introduction

Alzheimer’s disease (AD) is a neurodegenerative condition influenced by both genetic (approximately 70%) and environmental (around 30%) factors. Genetic contributors include genes linked to familial Alzheimer’s disease (FAD) and sporadic Alzheimer’s disease (SAD). Specifically, the APP (amyloid precursor protein), PSEN1 (Presenilin 1), and PSEN2 (Presenilin 2) genes are associated with FAD, while the APOE gene is linked to the sporadic form of the disease [1]. 

The major hallmarks of AD pathology are the extracellular buildup of β-amyloid (Aβ) plaques and the intracellular aggregation of neurofibrillary tangles (NFTs), which are composed of hyperphosphorylated tau. Additionally, AD is characterized by the loss of synapses and neurons, as well as gliosis [2]. The onset of Aβ pathology in individuals predisposed to AD occurs approximately 15–20 years before the anticipated onset of progressive cognitive decline. Elevated cerebrospinal fluid (CSF) tau levels indicate neuronal injury and correlate with disease severity [3].

The AD affects 5% to 11% of individuals over the age of 65 and may impact up to 40% of those aged 85 and older [4]. It has multiple clinical manifestations and co-existing pathologies such as corticobasal syndrome and dementia with Lewy bodies. A key challenge of AD, and often the primary concern, is memory impairment. Memory difficulties can diminish a person’s ability to live independently and are frequently cited as a reason for nursing home admission [5]. Also, AD is characterized by other cognitive impairments such as a reduction in attention, which can influence well-being and mood, and leads to a reduction in their quality of life. Except cognitive impairments, patients with AD and its pathologies may also present psychiatric symptoms, including visual hallucinations [6].

In recent years, there has been growing evidence that this cognitive decline in older adults with AD or dementia results in the deterioration of gait patterns [7]. There is evidence suggesting that, in addition to the connection between cognitive decline and motor deterioration, neuropsychiatric symptoms—such as increased agitation, delusions, and hallucinations—are also associated with functional decline [8].

Although gait performance decline is typically observed in the later stages of dementia, it may manifest much earlier. There is a link between gait disorders and the disruption of the highest levels of gait control. This is often associated with a deterioration in the individual’s previous level of function, reduced physical activity, and a decline in functional status in daily life [9]. This reduction in their quality of life may also provoke emotional issues such as depression and anxiety. These symptoms impair more of everyday life. As a result, AD degenerates an adult’s learning and reasoning ability and performing out daily activities.

Complementary techniques have been used in physiotherapy rehabilitation, and mental imagery (MI) is one of these techniques. MI is the mental rehearsal of a given action without its actual execution. Studies indicate that MI is beneficial in improving skilled performance across both healthy individuals [8] and clinical populations [10]. MI has been shown to facilitate motor learning [11] and promote “neural plasticity” [12]. The brain enables itself to adapt its physical structure through repeated practice and experience.

Therefore, MI has been used in many scientific fields, such as psychology [13], motor learning and motor control [14], nursing [15], and sports [16]. Regarding its use in clinical populations, it has been applied in musculoskeletal [17,18] and cardiorespiratory pathologies [19] in children, adolescents [20], adults, and geriatric populations [21]. Also, it has been utilized in neurological rehabilitation, specifically in cases of stroke [22], Parkinson’s disease [23], and multiple sclerosis [24]. The use of MI enhances gait speed, gait performance, balance, cognitive function, daily living activities, and overall quality of life. MI is a non-invasive, safe, and cost-effective therapy that can also be conveniently practiced at home [25]. 

The content of MI session differs regarding the aim of applying the MI intervention program. Usually, for physiotherapy rehabilitation reasons, the MI session includes exercises or tasks or activities that the participant followed previously. Research suggests that MI can enhance skilled performance in both healthy individuals [11] and clinical populations [10]. MI has been demonstrated to support motor learning [26] and foster “neural plasticity” [12]. It allows the brain to modify its physical structure through consistent practice and experience.

One of the key challenges in utilizing MI practice is ensuring that the patient remains engaged and focused enough to effectively perform MI exercises. Despite the widespread use of specific MI questionnaires and diaries in confirming patient’s vividness and controllability of images, in recent decades, new approaches have been established to confirm MI. A direct non-invasive approach involves evaluating the modulation of brainwaves recorded through continuous electroencephalography (EEG) monitoring during MI practice within the framework of a brain–computer interface (BCI). Thus, MI-based brain–computer interfaces (MI-BCIs) detect brain activity patterns related to the mental simulation of movement and translate them into commands for external devices. While the use of EEG-based BCI systems has been studied extensively in healthy individuals [27], their application in Alzheimer’s disease remains largely unexplored.

A study examined a therapeutic intervention using a non-invasive neuromodulation protocol in a 70-year-old female patient. They were diagnosed with corticobasal degeneration involving transcranial direct current stimulation (tDCS), action observation, and MI. The results showed positive effects on balance, functional strength, attentional set-shifting, working memory, and emotional well-being [28]. 

The present review describes the use of MI on Alzheimer’s disease-related dementia. We present several studies published from January 2020 to December 2024 on people who suffer from dementia, AD, or mild cognitive impairment (MCI). There have not been any published reviews examining the use of MI in older adults with dementia. In particular, no previous studies have investigated the effect of MI on dementia’s symptoms such as cognitive and motor problems that impair everyday life. Thus, we present the effects of MI in the motor, cognitive, and psychological state and quality of life of people with Alzheimer’s disease-related dementia. 

## 2. Methods

The present literature review describes the use and effectiveness of MI on Alzheimer’s disease-related dementia. A comprehensive search was carried out in seven databases, i.e., PubMed, Cochrane Central Register of Controlled Trials (Cochrane), Excerpta Medica Database (EMBASE), Scopus, Cumulative Index to Nursing and Allied Health Literature (CINAHL), Psychological Abstracts (PsycINFO), and Web of Science. These databases were utilized to conduct an extensive search of published studies, covering the period from January 2010 to December 31, 2024. The published studies were in the English language for ease of identifying relevant articles. We reviewed the titles and abstracts and evaluated them for inclusion. The inclusion criteria were as follows: (a) older adults with dementia, MCI, AD, (b) qualitative, quantitative, and mixed-method studies published in full text in English and open-access articles, (c) the usage of MI, (d) male and female older adults, and (e) the measurable variables in the studies that examine the use of MI include at least one of the following: balance, functional status, strength, cognitive ability, depression, anxiety, and quality of life. The exclusion criteria were as follows: (a) older adults with other neurological or neurogenerative diseases such as Parkinson’s and multiple sclerosis and (b) included other complementary techniques such as yoga, tai chi, etc. Two independent researchers meticulously applied the inclusion and exclusion criteria to all studies. The final selection of the studies to be included was collectively decided by all co-authors. The search included a combination of keywords mainly focused on Alzheimer’s disease, dementia, MCI, imagery, neurophysiological intervention, balance, functional status, strength, agility, cognitive ability, attention, memory and autobiographical memory, depression, anxiety, self-esteem, emotional status, psychological status, and quality of life. Also, we used key words with a certain combination using Boolean operators such as “Alzheimer AND imagery AND rehabilitation”, “dementia AND imagery AND memory”, and “dementia AND imagery AND balance”. Editorials and letters to the editor were not considered. 

As a result, this review incorporates studies that examine the application and effectiveness of MI in the rehabilitation of Alzheimer’s disease-related dementia. Examining the effect of MI on dementia has theoretical and clinical significance because this knowledge might help neurologists and physiotherapists include new methods and techniques in their therapeutic protocols in order to cope with the problems that dementia causes. The present review will contribute to adding research about the effect of complementary techniques such as MI on a neurodegenerative disease such as Alzheimer’s disease-related dementia. 

## 3. Discussion 

The discussion part of the present review contains three topics where each topic examines the effect of MI on the (a) cognitive function, (b) motor function, and (c) emotional function of older adults with Alzheimer’s disease-related dementia. Thus, each part described the results of studies on these topics.

### 3.1. Cognitive Function

AD is the most prevalent cause of dementia in older adults. People typically experience a transitional phase between normal aging and the onset of clinically evident AD. This transitional stage, referred to as MCI, is primarily marked by a decline in memory with little or no impact on functional abilities. Early signs of AD often involve impairments in overall functioning, episodic memory, processing speed, and executive abilities [29]. The initial clinical manifestations of MCI and AD are typically episodic memory impairments and disruptions in the attentional regulation of executive processes. Compared to healthy controls, individuals with AD show significantly reduced ability to recall physical attributes and semantic biographical details about well-known figures [30]. 

Patients with AD show a diminished ability to mentally visualize past and future events. Visual cues may enhance the specificity of memory during retrieval in AD. In particular, they may reduce the executive function demands that are necessary for voluntary recall [31]. The neurological damage associated with AD is linked to a reduced capacity to generate visual images. The parahippocampal cortex plays a role in scene processing [32]. Research indicates that damage to or near the parahippocampal area impairs the ability to recognize scenes or landmarks [33]. Patients with such neurological damage can map out locations they learned before the damage occurred, but not afterward. This suggests the parahippocampal area is crucial for encoding, rather than storing, scene-related information [34]. The hippocampus and surrounding regions are vital for binding visual details during recall [35]. Therefore, damage to the hippocampal and parahippocampal regions, as observed in AD [36], likely contributes to the inability to construct visual images [37]. Recent neuroimaging studies also show that activity in the primary visual cortex is associated with spontaneous memory-related visual imagery during word encoding. These findings are particularly relevant for AD patients. As primary visual areas are typically affected later in the progression of the disease compared to temporoparietal or prefrontal regions [38].

Indeed, visual imagery plays an important role in the memory system, particularly in the experience of memory, reliving in AD [39]. An association between autobiographical memory and the generation of mental images in AD is proposed. Autobiographical memories are commonly experienced as visual images. These images serve as the primary format for the subjective experience of such memories [40]. Autobiographical memory refers to a system that includes recollections from an individual’s life, integrating both episodic and semantic memory. It makes a type of explicit memory. Although explicit memory generally deteriorates in individuals with dementia, implicit memory—associated with unconscious learning—often remains preserved.

This preservation applies to implicit memory linked to sensory or motor learning, as opposed to conceptual learning. Research has shown that autobiographical memory and visual imagery are preserved, although spatial imagery may be impaired in AD. A notable connection was found between autobiographical memory and both visual and spatial imagery in participants with AD and controls. However, autobiographical memory was more closely predicted by visual imagery than by spatial imagery. The ability to access visual imagery and manipulate mental images in spatial contexts might be linked to autobiographical recall in AD. Specifically, visual imagery may aid in retrieving autobiographical memories in AD participants. It provides visual cues that facilitate quicker and easier navigation through the hierarchical structure of autobiographical memory [39,40]. Visual mental images assist in autobiographical recall by streamlining access to the hierarchical structure of these memories [41,42]. Studies have demonstrated that individuals with strong visual imagery skills tend to have better autobiographical recall than those with weaker visual imagery abilities. This highlights the significant role of MI in autobiographical memory [43].

This study showed that visual MI is compromised from the early stage of AD. The ability to generate visual images was selectively impaired in patients with MCI. Deficits in visual mental imagery are evident during the prodromal phase of AD [44]. Patients with mild AD were able to perform basic visual imagery tasks but were unable to effectively engage in more complex imagery tasks due to deficits in semantic memory, visuospatial abilities, and executive functions. As a result, individuals with AD can successfully perform basic MI. Hussey et al. [38] suggest that deficits in episodic memory may hinder AD patients from storing or retrieving general mental images created during encoding. The inability to perform more complex forms of MI in AD is attributed to neurocognitive impairments, which makes use of it potentially beneficial for improving memory in this population [38]. 

The relationship between the vividness of visual imagery and visual hallucinations in patients with AD was investigated [45]. AD patients have shown a significant positive relationship between visual hallucinations and the vividness of visual imagery. No significant relationship was found between auditory hallucinations and the vividness of visual imagery in these participants. Hallucinations occurred due to the vividness of visual imagery. These findings show a close relationship between the occurrence of visual (but not auditory) hallucinations and the generation of vivid visual images in AD [45].

So far, only a limited number of studies have explored the role of motor representations and their influence on perception and cognition in AD. In individuals with AD, the conscious visualization of body movements or the movement of visual stimuli takes significantly longer, without encountering particular difficulty in the motor condition. The rotation angle of visual stimuli in the mental hand rotation task affects the response time in AD patients. In these patients, response times in the reachability judgment task are notably prolonged, especially for stimuli located near the boundary of peripersonal space. AD patients experience a decline in implicit MI abilities, though explicit MI abilities remain intact. This decline impacts processing time, but not the accuracy of performance in motor-related perceptual and cognitive tasks [46].

Regarding the application and efficacy of MI in MCI or AD, a combination of MI and action observation therapy was applied in a sample of 30 patients with vascular cognitive impairment over an 8-week period. The participants underwent cognitive function assessments at baseline, after 4 weeks, at the 8-week mark post-treatment, and 1 month after the follow-up. Memory issues in vascular cognitive impairment tend to be less severe than in Alzheimer’s disease-related dementia. The results suggested that integrating cognitive training with MI and action observation therapy can positively influence cognitive function. Therefore, MI has been demonstrated to improve memory in older adults with mild AD [47]. Furthermore, a randomized controlled trial found that a 12-week MI program combined with the Otago exercise program in people with dementia led to improvements in their cognitive status compared to the two control groups [48]. Chistakou et al.’s study [46] is the first study that examined the kinesthetic type of MI, and not the visual or spatial imagery, on older adults with dementia. The limitations of Christakou et al.’s study [48] include the participants’ diverse ages, sexes, and specific stages of dementia. More research should replicate these findings because the importance of such findings may contribute to new treatment options for older adults with dementia (Table 1).

Regarding the memorization of intentions, MI has been strongly linked to prospective memory. Through MI, individuals simulate or recreate perceptual experiences, allowing them to perceive things that are not currently present. MI enhances performance throughout the stages of intention formation, retention, and execution. By thinking through and planning the details of an intention, individuals can encode it and strengthen the association between cues and context by pre-experiencing the specific visual–spatial environment where intention will be carried out. This process leads to improved prospective memory performance. Older adults with MCI exhibited significantly poorer performance during the intention formation phase. Also, they showed reduced performance in the intention retention phase, when compared to individuals experiencing normal aging. 

However, the use of MI significantly improved the performance of older adults with MCI across all phases of prospective memory, except for one [49].

**Table 1:** Summary of studies on mental imagery in dementia and Alzheimer’s disease.

Authors/Year	Purpose	Study Population	Design/Intervention	Reported Outcomes	Results	Limitations
Heyn [50]	To assess a multisensory exercise program’s impact on cognitive function, mood, and physical health	• 13 nursing home residents with moderate to severe AD (aged 70–93, mean = 85.7 ± 6.5, MMSE = 7.25 ± 3.4) • No control group	• Group intervention study • Multisensory exercise program including warm-up, flexibility, aerobic exercises, strength training, and relaxation techniques	• Resting heart rate • Blood pressure • Weight • Mood • Agitation	• Improvement in resting heart rate and mood • Increased engagement in physical activity	• Absence of a control group • Small sample size • Mood questionnaire requires further validation
Hussey et al. [36]	To examine whether mental imagery improves memory in mild AD and explore cognitive demands of imagery tasks	• 15 patients with mild AD (mean age = 77.5 ± 6.3, MMSE = 25.4 ± 2.6) • 15 healthy controls (mean age = 75.5 ± 7.9, MMSE = 29.3 ± 0.8)	• Intervention study • Participants completed four cognitive tasks (Clock Angles, Animal Tails, Taller/Wider, and Letter Curves tasks)	• Recognition performance • Impact of imagery on task engagement and memory	• Limited benefit of imagery in memory for AD patients • Basic visual imagery remained intact in AD patients	• Similar tasks for patients and controls could lead to ceiling/floor effects • Small sample size
Liu et al. [45]	To evaluate combined motor imagery and action observation therapy’s impact on vascular cognitive impairment	• 30 participants with vascular cognitive impairment • Experimental group: cognitive training + motor imagery and action observation therapy • Control groups: cognitive training only and motor imagery/action observation therapy only	• Randomized pilot study • Intervention lasted 8 weeks with evaluations at baseline, 4 weeks, 8 weeks, and 1-month follow-up	• Montreal Cognitive Assessment Scale • Rivermead Behavioral Memory Test	• Combination therapy showed improvements in cognitive function	• No true control group • Need for more validated assessment tools
Christakou et al. [46]	To study the effect of mental imagery on emotional, cognitive, and quality-of-life outcomes in early-stage dementia	• 160 participants (43 men, 117 women; MMSE = 23.2 ± 0.15) • Intervention: MI and exercise group • Control groups: exercise only or no intervention	• Randomized control trial • 24 sessions over 12 weeks (45 min/session) • Assessments at baseline, mid-intervention, and post-intervention; MI sessions included a 30-min guided imagery exercise following physiotherapy	• Walking Talking Test • Geriatric Depression Scale • EuroQol-5D scale	• MI positively impacted emotional state, cognitive abilities, and quality of life	• Heterogeneity in participant characteristics • Limited to early-stage dementia • Dropout rates
Christakou et al. [51]	To evaluate mental imagery’s effect on motor function, balance, and functional status in early-stage dementia	• Same as above	• Same as above •Assessments included functional tests like the Berg Balance Scale, Timed Up and Go test, and Functional Gait Assessment	• Berg Balance Scale • Functional Gait Assessment • Sit-to-Stand Test	• Improvements in balance and functional status	• Same as above

MMSE: Mini Mental State Examination, MI: Mental Imagery.

### 3.2. Motor Function 

Neurophysiological activity during MI closely mirrors that observed during actual movement execution. It remains unclear whether MI directly or indirectly corresponds to the motor process itself. The similarity between mental representations of motor actions and the actual muscle activity suggests that MI could trigger changes in the motor cortex, potentially influencing subsequent physical performance. During MI, the motor cortex exhibits the same overall population dynamics as during motor execution, reorganizing motor output and/or feedback components into a distinct, output-null imagery subspace [52]. The brain regions activated during MI overlap with those involved in motor execution [53], such as the premotor cortex, primary motor cortex, posterior parietal cortex, and the cerebellum [13]. The neural signals transmitted to the central nervous system are influenced by MI. The cognitive programming of motor responses occurring at the highest levels of the central nervous system is guided by the MI process. That makes MI responsible for adaptive changes in central processes. Stimulating the frontal lobe to enhance neural activity can potentially aid in acquiring MI skills, which could benefit neurological rehabilitation for individuals with MCI [54].

The onset and gradual progression of motor impairments throughout the course of AD has been examined [55]. These motor deficits may emerge following the cognitive symptoms of AD and could potentially increase the risk of developing the condition [35]. In individuals with MCI and AD, these motor changes are primarily associated with specific aspects of gait and balance function [56]. Additionally, they impair the execution of coordinated movements [57], which involves several biomechanical capacities required to perform actions. Also, they impair the neuronal processes responsible for planning, programming, and controlling those movements [58].

This study showed that AD patients have longer motor response times than healthy controls through a set of complementary motor-related perceptual and cognitive task while maintaining the same level of accuracy. Longer times necessary for AD patients to respond in these MI tasks seem to indicate that impairments in motor processes also have an impact on perception and cognition. The motor deficits in AD patients seem enhanced in the case of perceptual or cognitive tasks relying on implicit motor imagery [46].

Individuals with AD may experience deficits in postural and motor control, as the decline in gait performance can be detected even before the prodromal stage of MCI. There is a significant correlation between gait disorders and impairment in higher-level gait control [9]. Gait variability reflects the functional status of these higher-level control mechanisms. Individuals with AD exhibit lower gait variability compared to those with MCI. Early dysfunction in the cortical sensorimotor control of gait, particularly involving the hippocampus, contributes to this variability in individuals with MCI [9]. Additionally, between sixty and eighty percent of people with dementia experience falls each year [59]. Fall rates are higher among those with dementia compared to adults without dementia [60]. The early or mild stages of dementia are linked with osteoarthritis [61], pain [59], and poor balance [62], representing a critical time for early intervention. Another study reports that we can use an imagery version of the Timed Up and Go test (TUG) to assess any cognitive impairment for the first time. By using the time difference between a real (TUGr) and an imagined (TUGi) TUG task, Rüdiger et al. [50] examined the effect of cognitive impairment on the motor imagery ability of 52 participants with MCI. The main outcome of their study was the time difference between the TUGr and the TUGi. When the cognitive function decreased, the difference between TUGr and TUGi performance time and the TMT B performance time increased. The combination of TUGr and TUGi may contribute to assessing cognitive impairment, which is a possible pre-stage of dementia [51].

MI is employed to assess the most advanced level of gait control. Cognitive abilities, especially executive functions, influence the temporal alignment between actual movement and the mental simulation of gait in patients with conditions like dementia, schizophrenia, and multiple sclerosis, using a mental chronometry approach [9]. Beauchet et al. [57] indicated that the MI of gait could serve as a potential biomarker for MCI in older adults. Anderson et al. [51] highlighted that only a limited number of studies have explored the benefits of MI for individuals with dementia. Notably, Heyn [63] reported improvements in heart rate, mood, and physical exercise participation following a multisensory intervention combining imagery with a warm-up seated exercise session. Due to the scarcity of studies examining MI in older adults with dementia and AD, this technique currently holds an evidence grade of C1 [51]. Christakou et al. [64] found that participants in the experimental group, who underwent 24 sessions of MI combined with the Otago exercise program over three months, showed improved balance and functional status compared to the two control groups. These improvements were measured using the Time Up and Go test, Time Sit to Stand test, forward and lateral direction tests, the Functional Gait Assessment, and the Berg Balance Scale. Christakou et al.’s study is the only RCT that examines the use of kinesthetic MI with a large sample of individuals with dementia, and their results confirm the positive effect on their motor ability (Table 1). 

### 3.3. Emotional and Psychological State and Quality of Life 

Lyketsos et al. [65] reported that a large number of older adults with dementia will exhibit behavioral and psychological symptoms (BPSDs). Depression, anxiety, and apathy appeared in those with mild dementia at 38%, 41%, and 37%; moderate dementia at 38%, 41%, and 37%, respectively; and severe dementia at 54%, 59%, and 43%, respectively [66]. Mood disturbances such as agitation, reduced self-esteem, aggression, eating disturbances, and night-time behavior were also observed in AD patients [65]. 

Depression is a commonly investigated symptom in older adults with dementia. It is a severe mental condition characterized by persistently low mood, anhedonia, changes in appetite, sleep disturbances, and feelings of worthlessness. Depression has been associated with a higher rate of conversion from MCI to AD [67] and is prevalent among older adults with MCI [68]. Werner et al. [69] noted that dementia-related anxiety has primarily been studied in cognitively healthy, community-dwelling adults. Anxiety affects one-third of residents in long-term care facilities [70], influencing both their quality of life and care management. Additionally, attention decline is an early symptom of dementia, contributing to its progression, difficulties in performing daily activities, and communication disruptions. This decline may also be linked to impaired balance, increasing the risk of falls. Poor balance is frequently associated with cognitive impairment, which results from damage to the cerebral cortex that affects motor functions. However, research on attention deficits in dementia remains limited [71]. Further studies are needed to assess attention deficits with valid tools and to develop rehabilitation protocols to address these deficits in this clinical population.

Quality of life is a significant factor for older adults with dementia. It is influenced by the type and severity of dementia (i.e., early, middle, and late stage), the care provided by the rehabilitation personnel (i.e., physiotherapist, nurse, occupational therapist, etc.), and the individual’s personality (i.e., active, apathetic, motivated, anxious, depressive, etc.) prior to the onset of the condition. A key determinant of quality of life across all stages of dementia is the person’s mood [72]. Mood encompasses psychological well-being, depression, anxiety, happiness, self-esteem, and life satisfaction, which all influence quality of life [73]. Depression and anxiety in individuals with dementia are linked to lower quality of life [74]. Better quality of life is often observed in those with fewer depressive symptoms, irritability, and apathy [75]. Disruptions in mobility and social support negatively affect the quality of life of patients with dementia. Improved quality of life is more strongly associated with social interactions [76], better general health [77], and reduced caregiver burden [78].

The impact of MCI on emotional well-being, attention, and QOL in MCI or AD has been explored in a limited number of studies. Heyn [63] found a statistical improvement in overall emotional mood in a small sample of 13 individuals with mild AD, following an intervention that combined imagery with seated exercise. This intervention improved the emotional stability of older adults with mild AD. However, the study had limitations, such as the absence of a control group, lack of randomization, use of a small convenience sample, and instruments that required further validation. Hussey et al. [38] demonstrated that MI might help adults with mild AD address attention deficits and improve memory function. Although they found statistical significance, the study had limitations, including a small sample size (19 participants) and a lack of functional activities of daily living. Christakou et al. [48] showed that MI combined with the Otago exercise program improved quality of life, reduced depression and anxiety, and enhanced cognitive status, compared to two control groups. A major strength of this randomized controlled trial (RCT) was its large sample size, the use of valid tests for subjective evaluation, and, most importantly, the novel combination of the Otago exercise program with MI in older adults with dementia. This pioneering study opens new avenues for dementia treatment, offering rehabilitation personnel insights into this advantageous neurophysiological approach to MI (Table 1).

However, MI cannot be applied when the participant (a) has serious psychological/emotional issues (i.e., depression/anxiety), (b) is in a later stage of dementia, (c) has other neurosurgical health problems, and (d) cannot follow orders during the MI intervention. Therefore, it is important to assess participants’ ability to perform MI in the beginning with valid instruments as the Vividness of Visual Imagery Questionnaire, Kinesthetic and Visual Imagery Questionnaire, and Movement Imagery Questionnaire. These instruments should be validated in this clinical population. Lastly, it is crucial to examine the vividness and controllability of participant’s images during MI intervention after each session to ensure the effect of MI. 

## 4. Future Research Considerations

Few studies have been conducted on the effectiveness of MI on the motor and emotional state of Alzheimer’s disease-related dementia. The results of these studies proposed new treatment methods of dementia. Thus, they can help the rehabilitation personnel to apply more innovative techniques for their patients. More research should also investigate the effect of MI on other types of dementia. Future studies should investigate whether alterations in the central nervous system influence muscular, cognitive, and emotional states. If so, researchers should identify the specific neurophysiological mechanisms that contribute to the improvements in balance, functional performance, depression, and quality of life in individuals with dementia. Research should examine how the use of MI contributes to improving the motor learning processes of a task in this clinical population. Also, more research should examine if the MI technique allows the storage of new information in non-compromised cortical areas in patients with AD-type dementia. How the different types of memory, i.e., remote memory, may be improved using MI in this clinical population is a significant theoretical and clinical question that should be answered in future studies. Additionally, it is important to explore the impact of MI on other neurodegenerative conditions, considering other factors such as neuromuscular coordination and confidence. Furthermore, future research should incorporate more valid tests and/or instruments to assess the MI ability in this clinical population. In particular, specialized questionnaires are needed to evaluate different types of MI, like visual, spatial, and kinesthetic MI, in all types of dementia. Last, the rehabilitation personnel may increase the motor, emotional, and cognitive issues of older people with dementia by using ME. 

## 5. Conclusions

The influence of MI on the motor and emotional status of older adults with Alzheimer’s disease-related dementia has been examined in a few studies. In particular, the few existing findings showed a positive influence on the balance, functional status, depression, anxiety, and quality of life of older adults with dementia. Future studies should involve larger and more homogeneous samples, including participants of both sexes. The kinesthetic type of MI should be examined more in future studies as the majority of the existing results investigate visual or spatial imagery. Future research should investigate more variables such as strength, endurance, neuromuscular coordination, confidence, and kinesiophobia. Generally, due to the lack of studies and the limitation that these studies have, more research should be conducted on the effect of MI on older adults with Alzheimer’s disease-related dementia.
